# Pontine extension of a tentorial schwannoma without cranial nerve involvement: a case report

**DOI:** 10.1186/1752-1947-5-597

**Published:** 2011-12-28

**Authors:** Pietro Ivo D'Urso, Michele Marino, Arturo Di Blasi, Carmine Franco Muccio, Pompilio De Cillis, Giuseppe Catapano

**Affiliations:** 1 Neurosurgery Operative Unit, Department of Neuroscience, 'G Rummo' Hospital, Benevento, Italy

## Abstract

**Introduction:**

Intracranial schwannomas unrelated to the cranial nerves are uncommon. We report a new case of tentorial schwannoma unrelated to the cranial nerves, with extension into the pons. A literature review with discussion of the most relevant pathogenetic aspects is also performed.

**Case presentation:**

A 42-year-old Caucasian man was admitted with right-sided paresthesias and weakness of his upper and lower extremities. The neurological examination revealed right hemiparesis and hemi-hypoesthesia. A brain magnetic resonance imaging scan revealed a cerebellopontine lesion, arising from the left free edge of the tentorium, and extending into his pons. A piecemeal removal was performed through a retrosigmoid approach. The lesion was not found to be associated with any cranial nerves. The histological examination revealed a schwannoma Antoni type A. His postoperative course was uneventful. At one year follow-up, the patient was neurologically intact and the magnetic resonance imaging of his brain performed at that time showed complete removal without signs of recurrence.

**Conclusion:**

Tentorial schwannomas are rare clinical entities. Knowledge of their clinical, radiological and anatomical characteristics is very important for the correct diagnosis and management.

## Introduction

Schwannomas constitute about 8% of primary intracranial tumors and account for almost 80% to 90% of cerebellopontine angle tumors. Most cases are related to the eighth cranial nerve, and less commonly to other cranial nerves. Intracranial schwannomas not associated with cranial nerves are uncommon [1,2]. To date, only 11 cases of intracranial schwannomas arising from the tentorium have been reported [1,3-12].

We report a new case of a tentorial schwannoma unrelated to the cranial nerves, with extension into the pons. A literature review with the discussion of the most relevant pathogenetic aspects has also been performed.

## Case presentation

**Figure 1:**
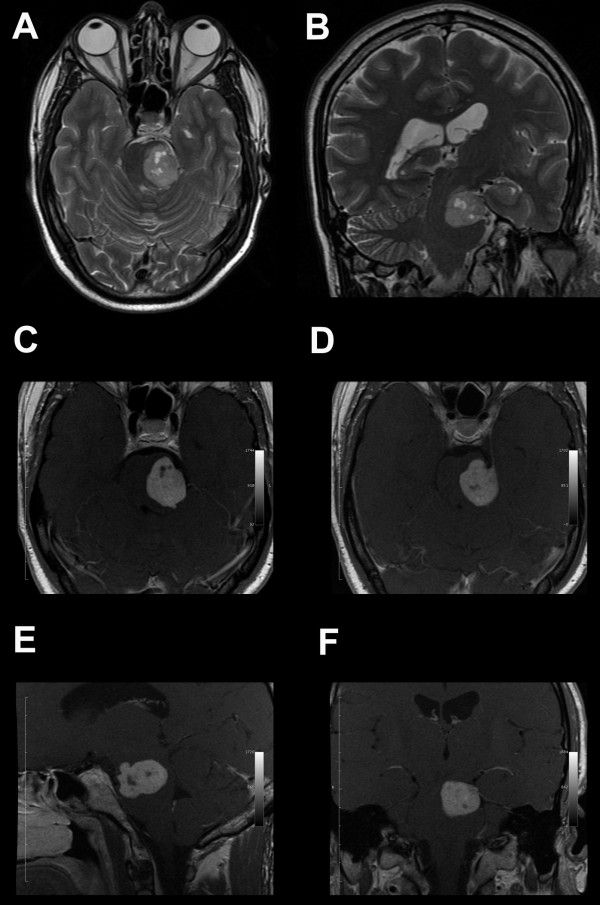
**Radiological findings. (A, B)**. T2-weighted magnetic resonance imaging scan showing a large hyperintense tentorial-based tumor with extension into the pons; microcystic areas are also visible inside the lesion: (A) axial; (B) coronal. (**C-F) **Post-contrast T1-weighted images demonstrating the gadolinium enhancement of the lesion - the dural tail sign and the tentorial attachment are also shown: (C) axial T1-weighted enhanced image; (D) axial T1-weighted enhanced image; (E) sagittal T1-weighted enhanced image; (F) coronal T1-weighted enhanced image.

A 42 year-old Caucasian man was admitted to our institution with a recent history of paresthesias and weakness of his right upper and lower extremities. The neurological examination revealed right hemiparesis and hemi-hypoesthesia. His cranial nerve functions were normal as was his brainstem auditory evoked response test. His personal and family histories were negative for neurofibromatosis. A brain magnetic resonance imaging (MRI) scan revealed a left cerebellopontine lesion, hypointense in T1-weighted images and hyperintense in T2-weighted images. A homogeneous contrast enhancement of the lesion was observed after gadolinium injection; the tumor appeared to be implanted on the left free edge of the tentorium, with extension into the pons. A 'dural tail' connecting the lesion to the tentorial edge was also present (Figure 1).

Surgery was performed using a retrosigmoid approach. After retraction of the hemispheric cerebellar lobe, a yellowish extra-axial lesion of the cerebellopontine angle was discovered. The tumor was strongly attached to the superior surface and the free edge of the tentorium, extending into the pons parenchyma. An endocapsular piecemeal removal was performed by means of an ultrasonic aspirator; the lesion appeared friable and vascularized. The cranial nerve complexes V, VII, VIII, IX, × and XI were all identified and preserved, and the mass was not found to be associated with these cranial nerves. The intraparenchymal portion was carefully dissected by the brain stem. After dissection from the surrounding structures, the lesion was mobilized and its implantation on the left free edge and superior surface of the tentorium exposed and resected. After tumor removal, the IV cranial nerve was exposed in its arachnoid cistern. At the end of the procedure, coagulation and resection of the implantation were achieved.

His postoperative course was uneventful and our patient fully recovered from his preoperative deficits.

**Figure 2:**
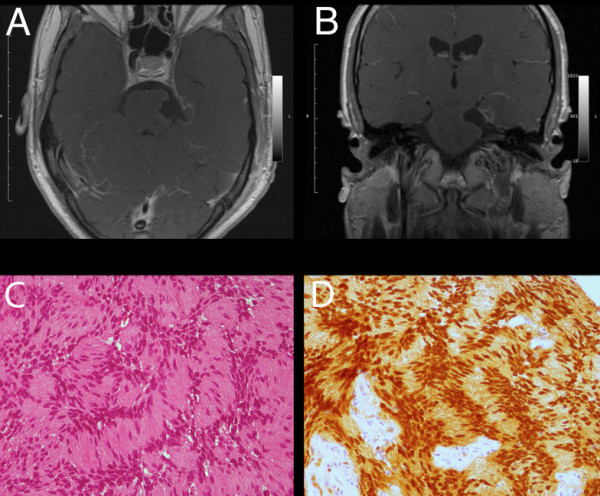
**Postoperative findings. (A, B)**. Postoperative T1-weighted enhanced images showing total tumor removal: (A) axial; (B) coronal. **(C) **Photomicrographs showing tissue formed by compact neoplastic proliferation of spindle-shaped cells, with elongated nuclei arranged in interlacing fascicles (hematoxylin and eosin; original magnification ×100). **(D) **Photomicrograph showing the tumor with strong positivity on immunostaining against S100 protein (original magnification ×100).

Histological examination of the resected tumor revealed schwannoma Antoni type A (Figure 2A-D). A brain MRI scan performed one year after the surgical procedure revealed total removal of the lesion, without signs of recurrence (Figure 2A, B). At that time, our patient continued to be neurologically intact.

## Discussion

Intracranial schwannomas unrelated to the cranial nerves are uncommon, since the central nervous system is devoid of Schwann cells. In 1966, Gibson published the first case of intracerebral Schwann cell tumor in the temporal lobe of a six-year-old boy [13]. Since that time, approximately 60 cases of schwannomas unrelated to the cranial nerves have been reported [14,15]. Most intracranial schwannomas unrelated to the cranial nerves have been described as being adherent to the dura mater and have been thought to arise from this structure [4,5,16-18]; in 10 cases, a tentorial attachment has been demonstrated (Table 1[1,3-11]). Interestingly, none of the patients of the reported cases had neurofibromatosis. Even in our case, the lesion arose from the tentorial dura, but an intraparenchymal extension into the pons was also present.

**Table 1:** Summary of the reported cases of tentorial schwannoma

Reference	*Age, sex*	*Presentation*	*Localization*	*MRI dural tail*	*Treatment*	*Results*	*Histology* *(Antoni's subtype)*	*Outcome *
[6]	22, M	Incidental	Left, transtentorial	-	Supra-infratentorial	Subtotal resection	Schwannoma (Antoni A)	Good
[7]	9, F	Dizziness, headache, vomiting, unsteadiness	Right, infratentorial	+	Retrosigmoid	Total resection	Schwannoma (N/A)	Complete recovery MRI: no residual/recurrent tumor (12 months)
[5]	41, F	Headache, vertigo. Truncal ataxia, unsteadiness	Left, infratentorial	+	Retrosigmoid	Total resection	Schwannoma (N/A)	Complete recovery MRI: no residual/recurrent tumor (16 months)
[8]	29, M	Headache, transient diplopia.	Right, infratentorial	+	Petrosal	Total resection	Schwannoma (Antoni A and B)	IV cranial nerve deficit (2 months)
[4]	17, F	Headache, diplopia, nystagmus	Left, infratentorial	+	Pterional	Total resection	Schwannoma (N/A)	Complete recovery MRI: no residual tumor
[1]	23, M	Dizziness, swallowing difficulty	Right, infratentorial	-	Petrosal	Total resection	Schwannoma (Antoni A)	Complete recovery
[3]	49, F	Headache, neck pain, vomiting, unsteadiness; right facial paresis; right facial paresthesia; neurofibromatosis	Right, infratentorial	+	Retrosigmoid	Total resection	Schwannoma (N/A)	Recovery, except for residual facial paresthesia (1 month)
[10]	20, M	Headache	Right,		Subtemporal transtentorial	Total resection	Schwannoma (N/A)	Good (at 6 months)
[9]	60, F	Headache	Infratentorial	+	Suboccipital	Subtotal resection	Schwannoma (N/A)	Good MRI: small residual tumor
[11]	64, F	Transient global amnesia	Right, supratentorial	-	Subtemporal	Subtotal resection	Schwannoma (N/A)	Good MRI: residual tumor
[12]	58, F	Headache	Infratentorial	+	Retrosigmoid	Subtotal resection	Schwannoma (Antoni A & B)	Good, stable residual tumor (24 months)

Present case	42, M	Paresthesia and weakness of the four extremities	Left, infratentorial	+	Retrosigmoid	Total resection	Schwannoma (Antoni A)	Complete recovery MRI: no residual/recurrent tumor (12 months)

F: female; M: male; N/A: not applicable; +/-: presence/absence of dural tail on magnetic resonance imaging (MRI).

Typically, tentorial-based schwannomas appear on radiology as a heterogeneous enhancing cystic lesion, occasionally encased in the surrounding brain parenchyma, but without peritumoral edema; as in our case, a dural tail sign is often present [5].

Several surgical approaches have been reported to remove this kind of tumor (Table 1). In our case, we used a retrosigmoid approach. An alternative approach could be a subtemporal route, because of the tumor location in the middle incisural space. However, dealing with a left-sided lesion in a right-handed patient, we preferred a retrosigmoid approach to avoid a temporal venous infarction secondary to brain retraction, a complication reported as symptomatic in 2.6% to 13% of these approaches [19].

There are several hypotheses about the origin of intracranial schwannomas unrelated to the cranial nerves [20]. Interestingly, some authors have suggested that these lesions could arise from Schwann cells normally present within the perivascular nerve plexuses surrounding arteries in the subarachnoid space, or from Schwann cells of meningeal branches of the trigeminal and anterior ethmoid nerves [21]. The absence of cranial nerve deficits and the frequent presence of a unilateral headache strongly suggest a possible origin of the thin branches of the trigeminal ganglion or of the sensorial branches of the first division of the trigeminal nerve which supplies the tentorium. These branches were first described by Arnold in 1826 [22].

Although the absence of postoperative cranial nerves deficits has been advocated as proof that these lesions do not arise from cranial nerves, this observation has been questioned by some authors, since a lack of postoperative symptoms could be related to the compensative mechanism of ocular movements [4].

## Conclusions

Tentorial schwannomas are rare clinical entities, but the knowledge of their clinical, radiological and anatomical characteristics is very important for a correct diagnosis and management.

## Consent

Written informed consent was obtained from the patient for publication of this manuscript and any accompanying images. A copy of the written consent is available for review by the Editor-in-Chief of this journal.

## Competing interests

The authors declare that they have no competing interests.

## Authors' contributions

DPI analyzed and interpreted the patient data, and was the major contributor in writing the manuscript. MM performed the surgical procedure. DBA performed the histological examination. MCF analyzed and interpreted radiological data. DCP analyzed and interpreted clinical data. CG performed the surgical procedure and critically reviewed the manuscript. All authors read and approved the final manuscript.

## References

[B1] Anton T Guttierez J Rock J Tentorial schwannoma: a case report and review of the literature J Neurooncol 2006 76 307 311 10.1007/s11060-005-7286-y 16200344

[B2] Bruni P Esposito S Greco R Oddi G Solitary intracerebral schwannoma in von Recklinghausen's disease Surg Neurol 1984 22 360 364 10.1016/0090-3019(84)90140-X 6433498

[B3] Chung KH Cherian M Chandran KN Schwannoma with tentorial attachment in the cerebellopontine angle mimicking a meningioma J Clin Neurosci 2007 14 797 801 10.1016/j.jocn.2006.05.008 17532220

[B4] Du R Dhoot J McDermott MW Gupta N Cystic schwannoma of the anterior tentorial hiatus. Case report and review of the literature Pediatr Neurosurg 2003 38 167 173 10.1159/000069094 12646734

[B5] Oikawa A Takeda N Aoki N Takizawa T Sakoma T Schwannoma arising from the tentorium at an unusual location: case report Neurosurgery 2002 50 1352 1355 12015855 10.1097/00006123-200206000-00028

[B6] Flickinger FW Yuh WT Sato Y Hart MN MR findings of an unusual intracranial neuroma simulating a meningioma J Comput Assist Tomogr 1988 12 485 488 10.1097/00004728-198805010-00025 3366967

[B7] Jabbour P Rizk T Lahoud GA Hourani R Checrallah A Samaha E Nohra G Moussa R Okais N Schwannoma of the tentorium cerebelli in a child. Case report Pediatr Neurosurg 2002 36 153 156 10.1159/000048371 11919450

[B8] Ozawa N Nakayama K Ohata K Okamura T Inoue Y Tentorial schwannoma: a case report Br J Radiol 2003 76 421 424 10.1259/bjr/20106153 12814930

[B9] Calisaneller T Ozen O Altinors N Caner H Tentorium schwannoma mimicking meningioma: an unusual location Turk Neurosurg 2008 18 316 319 18814126

[B10] Hayashi N Kurimoto M Nagai S Sato H Hori S Endo S Tentorial incision in a lateral-medial direction with minimal retraction of the temporal lobe in the subtemporal transtentorial approach to the middle tentorial incisural space Minim Invasive Neurosurg 2008 51 340 344 10.1055/s-0028-1085452 19061145

[B11] Nitta N Shiino A Ishida M Okabe H Nozaki K Tentorial schwannoma in a 64-year-old female: case report Neurol Med Chir (Tokyo) 2011 51 239 243 10.2176/nmc.51.239 21441745

[B12] Nagata T Goto T Ichinose T Tsuyuguchi N Ohata K Tentorial schwannoma mimicking meningioma Neurol Med Chir (Tokyo) 2011 51 382 385 10.2176/nmc.51.382 21613767

[B13] Gibson AA Hendrick EB Conen PE Case reports. Intracerebral schwannoma. Report of a case J Neurosurg 1966 24 552 557 10.3171/jns.1966.24.2.0552 5935382

[B14] Haga Y Shoji H Oguro K Mori S Kawai T Shinoda S Masuzawa T Saito K Intracerebral Schwannoma--case report Neurol Med Chir (Tokyo) 1997 37 551 555 10.2176/nmc.37.551 9259156

[B15] Huang PP Zagzag D Benjamin V Intracranial schwannoma presenting as a subfrontal tumor: case report Neurosurgery 1997 40 194 197 8971843 10.1097/00006123-199701000-00043

[B16] Frim DM Ogilvy CS Vonsattal JP Chapman PH Is intracerebral schwannoma a developmental tumor of children and young adults? Case report and review Pediatr Neurosurg 1992 18 190 194 10.1159/000120661 1472431

[B17] Ghosh S Chandy MJ Solitary ectopic intracerebral schwannoma Br J Neurosurg 1992 6 163 166 10.3109/02688699209002921 1590972

[B18] Horgan MA Kernan JC Delashaw JB Schwartz MS Kuether T Schwannoma of the torcula presenting as an occipital mass. Case illustration J Neurosurg 1998 89 490 10.3171/jns.1998.89.3.0490 9724130

[B19] Nakase H Shin Y Nakagawa I Kimura R Sakaki T Clinical features of postoperative cerebral venous infarction Acta Neurochir (Wien) 2005 147 621 626 discussion 626 10.1007/s00701-005-0501-y 15770350

[B20] Feigin I Ogata J Schwann cells and peripheral myelin within human central nervous tissues: the mesenchymal character of Schwann cells J Neuropathol Exp Neurol 1971 30 603 612 10.1097/00005072-197110000-00005 5135015

[B21] Nelson E Rennels M Innervation of intracranial arteries Brain 1970 93 475 490 10.1093/brain/93.3.475 4097005

[B22] Uryvaev MY Sudarikova TV Trufanov IN Gorskaya TV Tsybul'kin AG The fine branches of the human trigeminal nerve Neurosci Behav Physiol 2008 38 157 160 10.1007/s11055-008-0023-1 18197382

